# Self-related motivational aspects of hindsight bias in soccer athletes

**DOI:** 10.3389/fspor.2025.1629424

**Published:** 2025-11-27

**Authors:** Tianpei Li, Haifeng Guo, Jin Hwang

**Affiliations:** 1 College of Physical Education, Jeonbuk National University, Jeonju, Republic of Korean; 2 College of Physical Education, Henan University of Science and Technology, Luoyang, China

**Keywords:** hindsight bias, sports, self-related, soccer athletes, motivational (message) framing

## Abstract

**Purpose:**

Hindsight bias is known to hinder people from learning and make people too adventurous in future decisions. The influence of hindsight bias on athletes, however, has not been widely and extensively investigated. Thus, the purpose of this study is to examine self-related motivational aspects of hindsight bias in a sports context.

**Method:**

Questionnaires were completed by 84 soccer players in a retrospective-design study. Respondents were divided into winning team members vs. losing team members and playing members vs. nonplaying members to assess the culpability for a negative outcome and outcome controllability.

**Results:**

Two-way repeated-measures ANOVA revealed significant hindsight bias for the expected match score regardless of team and player factors. In terms of player factors, playing members of the losing team and nonplaying members of the winning team increased their confidence levels for the expected match score. In terms of team factors, winning team members increased their confidence levels for the expected winning team. The reverse was true for losing team members.

**Conclusions:**

Playing members of a losing team used retroactive pessimism and showed greater hindsight bias. Winning team members showed greater hindsight bias, consistent with previous findings that people show hindsight bias for their own good performances. The results are further discussed in light of outcome controllability, expertise, and disappointment.

## Introduction

The uncertainty of wins and losses in sports attracts fans, and this uncertainty is malleable. Spectators often believe that they were certain about a potentially unforeseeable match outcome once the match has ended, for example, “***I knew that our team would lose this match****”* or “***I knew that we would win this game because our fitness level was excellent****”.* This phenomenon can be empirically examined as *hindsight bias*, which has been conceptualized and theorized in social and cognitive psychology. Hindsight bias is defined as “***a tendency to exaggerate one's ability to have foreseen the outcome of an event, after learning the outcome***” (49, p. 190). People who display hindsight bias tend to overestimate or misjudge their ability to predict outcomes and deny that their opinions are influenced by what they knew after the fact (1). Given that hindsight bias is the difference between recalled likelihood estimates and original estimates and that hindsight bias is more often observed for unexpected outcomes (2), sports settings that provide uncertainty are useful settings for conducting research on hindsight bias.

Fischhoff (1) first introduced the concept of hindsight bias in his seminal article. Since then, researchers have studied this phenomenon by primarily focusing on the cognitive effects of gaining outcome knowledge and have posited that hindsight bias is a type of cognitive illusion (3). This cognitive illusion leads people to perceive events or facts to be more determined, inevitable, or foreseeable, or misremember their predictions (4). Creeping determinism, biased causal reasoning, and biased memory reconstruction processes have been employed to examine this phenomenon (50). More recently, it was found that self-related motivational processes play an important role in hindsight cognitions, especially when event outcomes are associated with the self and arouse self-defensive processes (5). Nevertheless, few published studies on sports have investigated if self-related motivational processes influence hindsight cognitions in sports settings. Thus, the main purpose of this study is to examine the role of self-related motivational processes in hindsight cognitions in sports settings.

In addition to satisfying theoretical interest, exploring hindsight bias in sports settings matters because it can have implications for educational practice (6) and for decision making (7). If athletes tend to have hindsight bias, they are likely to have difficulties assessing their rate of learning new skills. They may overestimate their previous skills and think they only have a little to learn, consequently employing less effort to learn new skills than in normal learning. Athletes' decision making is also influenced by hindsight bias wherein athletes may focus on the wrong causal inference or exaggerate a particular cause of an outcome, resulting in myopic thinking and overlooking other perspectives when making future decisions. Hindsight bias also makes people overly risky in their future decisions (8). In the sections that follow, we briefly review empirical and theoretical work on hindsight bias and provide our rationale for the present study design.

### Cognitional approach

Hindsight bias research can be generally divided into two different approaches: a cognitional approach and a motivational approach. The two approaches view the causes of hindsight bias differently and assume two different processes by which the bias comes about. The cognitional approach views hindsight bias as a robust phenomenon that persists even when people attempt to eliminate it or when they are warned about its effects because it is an automated cognitive process that people cannot control (9). As such, hindsight bias has been demonstrated in a wide array of domains and in different countries (10).

The cognitional approach explains hindsight bias through the lenses of creeping determinism, memory reconstruction, and causal reasoning. This perspective is grounded in the idea that memory processes play a crucial role in the development of hindsight bias (11). Fischhoff (1) proposed the term creeping determinism because the knowledge of the actual outcome of an unpredictable event produces deterministic explanations of the outcome, leading people to believe that they already knew the outcome of the event. He called this the “***knew it all along***” effect, often used to characterize a metacognitive state in hindsight subjects (12). People are typically unable to discern that their predictive judgments are altered by their retrospective judgments because the integration of the outcome information into people's cognitional structure is immediate and unconscious (1). Thus, in creeping determinism, hindsight bias occurs without effort and is essentially unavoidable.

Researchers favoring memory reconstruction and causal reasoning propose that the human memory system is designed to inaccurately reconstruct the past to better make sense of what has occurred (13). This adaptive process of knowledge updating contributes to improving causal inferences, creating reasons for the outcomes, and subsequently producing hindsight bias. This notion is further supported by the causal model theory (CMT), which posits that people create causal reasons between a starting event condition and a completed outcome, forming internal causality, and unconsciously or consciously advocate for the actual outcome to make sense of the occurrence of the outcome (14). This sense-making mechanism is thought to be the most common cause for the occurrence of hindsight bias (15). However, on occasions where hindsight bias does not occur, the cognitive approach is not a suitable explanation. For example, people in a presidential security office may notice that a bag looks strange but not consider it a threat. If that bag was later found to have a bomb, do these people claim that they knew it all along? These situations, with mostly negative outcomes, in which the bias does not occur, seem contradictory to the current trends in hindsight bias research (11). Currently, the alternative approach, the motivational approach, has been used to obtain a better understanding of the underlying mechanisms of these situations.

### Motivational approach

The motivational approach postulates that hindsight bias is fueled by a motivational input and self-esteem (16). People are motivated to preserve their intelligence self-image, referred to as *ego defense* (17), especially when an outcome has personal relevance to them. When talking about the past, people want to take credit for successes and attribute failures to others (18). For example, Louie et al. (19) demonstrated that MBA students showed hindsight bias for their own good performance or a competing team's failure but no bias for their own failures or a competing team's good performance. If an ego defense is not successfully executed, people invoke a self-defensive process in which they attempt to reduce their sense of culpability by denying the foreseeability of the negative outcome (20). For example, Mark and colleagues showed that people believe that the failure of a stock purchase is more unforeseeable than do opponents or observers who were not involved with the outcome. In such cases, people are likely to say, “***I would not know the event results***”, showing less hindsight bias. Motivational mechanisms have been mainly hypothesized to reduce the bias for a negative outcome, and this hypothesis has been supported in several previous studies (21).

However, explanations of self-related motivational processes are complicated by a set of studies conducted by Tykocinski and colleagues (22–24). These authors observed greater hindsight bias for a negative outcome and introduced the concept of *retroactive pessimism* to explain this phenomenon. They argued that people use retroactive pessimism as a means of coping with disappointment, such that the magnitude of hindsight bias is proportional to the degree of disappointment. People consider the chances of success for a very disappointing outcome to be much lower than that for a less disappointing outcome. If people are very disappointed in an outcome, they tend to consider the outcome inevitable because “an inescapable failure might be easier to digest than a failure that could have been easily avoided” (22). Thus, perceiving a negative outcome as inevitable may be a great strategy to address disappointment and may sometimes be more comforting. In this way, people tend to accept negative outcomes and show hindsight bias. To support this notion, Tykocinski demonstrated that respondents who were disappointed with the result of an election outcome considered the results to be more likely than did those who were pleased with the election. In a subsequent study, respondents showed greater hindsight bias when they expected to apply for a large college stipend than a smaller one [(23), Experiments 2 and 3].

In addition to the role of disappointment, retroactive pessimism is associated with the degree to which people feel a sense of responsibility for negative outcomes, since self-related motivational processes are initiated only if people feel a responsibility for negative outcomes (20). If people do not have culpability for negative outcomes, the negative outcomes do not threaten their self-esteem. When there is no need to implement self-defensive processes, people use retroactive pessimism, which creates hindsight bias.

In a similar vein, Blank et al. (25) introduced the ***foreseeability*** and ***inevitability*** components of hindsight bias to explain culpability for negative outcomes in divergent hindsight bias. The foreseeability and inevitability components refer to whether people consider event outcomes to be foreseeable or inevitable, respectively. Blank and colleagues explained that foreseeability is one's subjective state of knowledge at a given time, whereas inevitability reflects an objective state of the world. Foreseeable outcomes are essential components of the self-defensive process because these outcomes are associated with those who make predictions or decisions of outcomes, producing a sense of responsibility for the outcomes. In contrast, inevitability is a key component of retroactive pessimism because people can believe that there was objectively nothing they could have done to change outcomes. In this way, people can avoid being responsible for the outcomes. Blank and Peters (26) suggested that all the studies showing reduced hindsight bias for self-related negative outcomes essentially capture the foreseeability component of hindsight bias.

A key variable influencing disappointment, culpability, foreseeability, and inevitability in terms of hindsight bias is outcome controllability. Tykocinski and Steinberg (24) focused on the role of outcome controllability and argued: “***it is easier to conclude that ‘I never had a chance to succeed' when the negative outcomes are uncontrollable***” (p. 554). With low outcome controllability, people feel a low sense of responsibility for negative outcomes and are more likely to consider the negative outcomes to be inevitable. Consequently, retroactive pessimism occurs, and greater hindsight bias is produced. In contrast, high outcome controllability leads people to feel a great sense of responsibility for negative outcomes and consider the negative outcomes to be foreseeable. In such a situation, the self-defensive process is a likely choice for people, who subsequently show less hindsight bias. Tykocinski and Steinberg (24) demonstrated that retroactive pessimism occurred only for uncontrollable events. Thus, motivational approaches have been used for a better understanding of the hindsight bias associated with negative outcomes.

### Hindsight bias research in sports

Leary (27) explored football spectators' hindsight bias and found that the spectators' postgame estimates of what they would have predicted for a score were close to the actual score. He commented that the course of the game biased spectators' perceptions of what they knew prior to the game, creating hindsight bias. Bonds-Raacke, Fryer, Nicks, and Durr (28) demonstrated hindsight bias in football spectators of Super Bowl XXXIII and reported that respondents who were instructed on the effects of hindsight bias were still not immune to its effects. Sanna and Schwarz (29) reported that football spectators who generated more alternative outcomes displayed more hindsight bias than those who generated fewer outcomes. Pezzo (30) examined the relationship between ego-involvement and hindsight bias, using home-team spectators and away-team spectators. He reported that the magnitude of hindsight bias was influenced by the spectators' ego-involvement in the team they supported. With a Korean sample, Hwang and Kim (31) reported that spectators' predictions of the result of a match was reshaped not only after they knew the match outcome but also as they watch the match. Gray et al. (32) studied whether hindsight bias is different between expert and novice batters and reported that the expert batters showed less hindsight bias than did novice batters.

In summary, this literature review reveals that research on hindsight bias in sports is limited compared to other domains, and cognitive approaches have been predominantly used to examine hindsight bias in sports. Self-defensive processes and retroactive pessimism have addressed self-related motivational aspects of hindsight bias for negative outcomes, yet the predictions from these perspectives are opposing. For self-defensive processes, hindsight bias is attenuated, and in retroactive pessimism, the bias is accentuated. In general, greater hindsight bias is observed for positive outcomes, uncontrollable negative outcomes, or outcomes that produce great disappointment. In contrast, less hindsight bias likely occurs for controllable negative or less disappointing outcomes.

### Present study

The purpose of this study was to investigate the self-related motivational aspects of hindsight bias. For this purpose, the following presuppositions were required to be considered. First, participants must have self-relevance to the outcome so that they are motivated to preserve their intelligence self-image. Next, the outcome must be deemed either negative or positive. Lastly, the outcome must be considered either controllable or uncontrollable.

In this study, respondents were elite soccer players who presumably had self-relevance to their team's match outcomes. Respondents were divided into winning team members and losing team members because it was expected that the winning team members considered the match outcome to be positive and that the losing team members considered the match outcome to be negative. This design allowed us to investigate how a match outcome influences respondents' hindsight bias and whether respondents employ an ego defense for a positive outcome or a self-defensive process or retroactive pessimism for a negative outcome. Based on the literature, it was considered an indication of an ego defense if the winning team members showed greater hindsight bias, whereas if the losing team members showed greater hindsight bias, it was considered retroactive pessimism to cope with the disappointment of a negative outcome and a self-defensive process to cope with culpability for a negative outcome. Respondents were also divided into playing members and nonplaying members under the supposition that playing members might consider an outcome more controllable than nonplaying members might. This design allows us to assess how outcome controllability influences respondents' hindsight bias. In retroactive pessimism, nonplaying members on a losing team should show greater hindsight bias. For a self-defensive process, playing members on a losing team should show less hindsight bias.

## Method

### Participants

Male Korean soccer athletes (mean age of 21.7) from Jeonju (J) University (*n* = 43) and Woosuk (W) University (*n* = 41) participated in this study. Among the 84 participants, 16 of 41 players from J University and 18 of 41 players from W University were playing members. Historically, these two teams are competitive with each other, and J University has two wins and one loss against W University. Tabachnick and Fidell (33) suggested that for repeated measures ANOVA, the minimum sample size for each group is 10 plus the number of dependent variables. Based on this, the number of participants (*n* 16) available for each group in this study satisfied the minimum sample size requirement. Tables 1, 2 shows participants' characteristics. The research protocol was approved by the Ethics Committee of Zhengzhou University (**ZDLL—20250377**) and adhered to the ethical standards outlined in the 1964 Declaration of Helsinki and its later amendments. All participants signed an informed consent form.

**Table 1:** Participants from J university and W university.

Teams	Players	Playing members	Nonplaying members	Past matches
J Univ.	43	16	27	2 wins, 1 loss
W Univ.	41	18	23	1 win, 2 losses
Total	84	34	50	

**Table 2:** Means and standard deviations of expected match scores and expected winning team.

			Prediction		Retrospection	
	Teams	Players	M	SD	M	SD
Expected match score (%)	J team	Playing members (*n* = 16)	69.687	7.846	69.689	7.846
		Nonplaying members (*n* = 27)	67.037	7.240	68.148	7.357
	W team	Playing members (*n* = 18)	66.389	8.008	67.500	8.445
		Nonplaying members (*n* = 23)	63.478	5.727	63.478	5.728
Expected winning team (%)	J team	Playing members (*n* = 16)	70.313	6.447	71.563	6.250
		Nonplaying members (*n* = 27)	70.185	5.962	71.296	6.138
	W team	Playing members (*n* = 18)	68.333	7.071	65.000	7.071
		Nonplaying members (*n* = 23)	69.130	6.683	68.696	6.255

### Procedures and measures

Two basic designs, hypothetical and memory designs, have been used for most hindsight bias studies (34). In hypothetical designs, an outcome is given to participants, and then researchers ask the participants how they would predict the outcome as if they had not been provided with the outcome information. In memory designs, participants first make a prediction, and then they are provided with an outcome, and finally, they are asked to recall their prediction. For the current study, a memory design was used in which participants made predictive judgments before the soccer match (i.e., foresight) and were then asked to recall their predictive judgments after knowing the match outcome (i.e., hindsight). If significant differences between the predictive judgments and the retrospective judgments were found, the occurrence of hindsight bias was identified.

Ethical approval was obtained, and informed consent forms were collected from all the participants. One hour before the soccer match, all soccer athletes were asked to make their predictions about the expected winning team [(23, 27, 35), Experiment 1]. Every participant indicated that his team would win over the other team using foresight. Then, participants rated their confidence level in the expected winning team in a range from 0% to 100% and answered the following questions for the foresight measurement:

Which team do you expect to win the match?

How confident are you making a correct prediction about the expected winning team? Please write any number between 0% and 100% for your confidence rating.

Four days after the soccer match, participants were asked to recall their expected winning team and confidence ratings. The recalled predictions and confidence ratings were measured four days later because Creyer and Ross (36) suggested that hindsight bias can be accurately measured three to seven days after an events. Participants read the following during the measurement of hindsight bias:

These questions are designed to measure how accurate your prediction was before the match. Please suppose that you do not know the match outcome and start thinking about the prediction you made before the match as accurately as you can. Then, please answer the following questions:

Before the match, which team did you expect to win the match? Before the match, how confident were you in making a correct prediction about the winning team? Please write any number between 0% and 100% for your confidence rating.

In addition to these questions, participants were also asked to indicate whether they were chosen to play or if they were benchwarmers in the soccer match. If participants were chosen to play the match, they were categorized as playing members regardless of the amount of time that they played.

In hindsight, every participant identified that his team would win over the other team, just as in foresight. Then, participants rated their confidence levels in the expected winning team, corresponding to the team they indicated. For data analysis, confidence ratings were only used to measure participants' foresight and hindsight, as Powell (37) stated that foresight-hindsight differences were specifically elucidated for confidence ratings. The means and standard deviations of the confidence ratings for the expected winning team are presented in Table 3.

**Table 3:** The means and standard deviations of the confidence ratings for the expected winning team are presented.

	Source	Type III sum of squares	df	Mean square	*F*	Sig.	$\eta_{p}^{2}$
Expected match score (%)	PR	12.435	1	12.435	4.476	.037*	.053
	PR × Team	.000	1	.000	.000	1.000	.000
	PR × Member	.000	1	.000	.000	1.000	.000
	PR × Team × Member	12.435	1	12.345	4.476	.037*	.053
Expected winning team (%)	PR	4.985	1	4.985	.459	.500	.006
	PR × Team	94.595	1	94.595	8.712	.004**	.098
	PR × Member	19.176	1	19.176	1.766	.188	.022
	PR × Team × Member	23.231	1	23.231	2.139	.147	.026

*Significance at *p* < 0.05.

**Significance at *p* < 0.01.

#### Data analysis and results

This study explored how soccer athletes' propensity for hindsight differs according to team (winning team vs. losing team) and player (playing members vs. nonplaying members) factors. To do so, this study used a 4 (team and player factors: winning team playing members, winning team nonplaying members, losing team playing members, and losing team nonplaying members) 2 (time factor: foresight and hindsight) design to analyze the confidence ratings for the expected winning team. In this design, time was a two-level within-subjects factor and the teams and players were four-level between-subjects factors (i.e., repeated-measures ANOVA with between-subjects factors). SPSS was used for data analysis.

The J team won the match over the W team by a score of 1–0. The game was competitive, and a late penalty kick by the J team resulted in the W team's loss. The results revealed significant differences in the confidence ratings for the expected winning team between foresight and hindsight.

### Expected winning team

Levene's tests for the repeated-measures variables for confidence ratings for the expected winning team for foresight, *F*(3, 80) = .70, *p* = .56, and hindsight, *F*(3, 80) = .74, *p* = .53, were nonsignificant. These tests showed that the variances were homogeneous for all levels of the repeated-measures variables and indicated that the *F*-tests for repeated-measures variables are reliable (38). This study had two within-subjects conditions (time factor: foresight and hindsight). Thus, Mauchly's test to examine violations of sphericity was not conducted because at least three conditions are necessary for sphericity to be a concern. The main effect of the confidence ratings for the expected winning team between foresight and hindsight was nonsignificant, *F*(1, 80) = .46, *p* = .50, = .006. This finding indicated that when team and player factors were ignored and only the time factor was considered, there was no identified hindsight bias in participants' confidence levels for the expected winning team.

**Figure 1:**
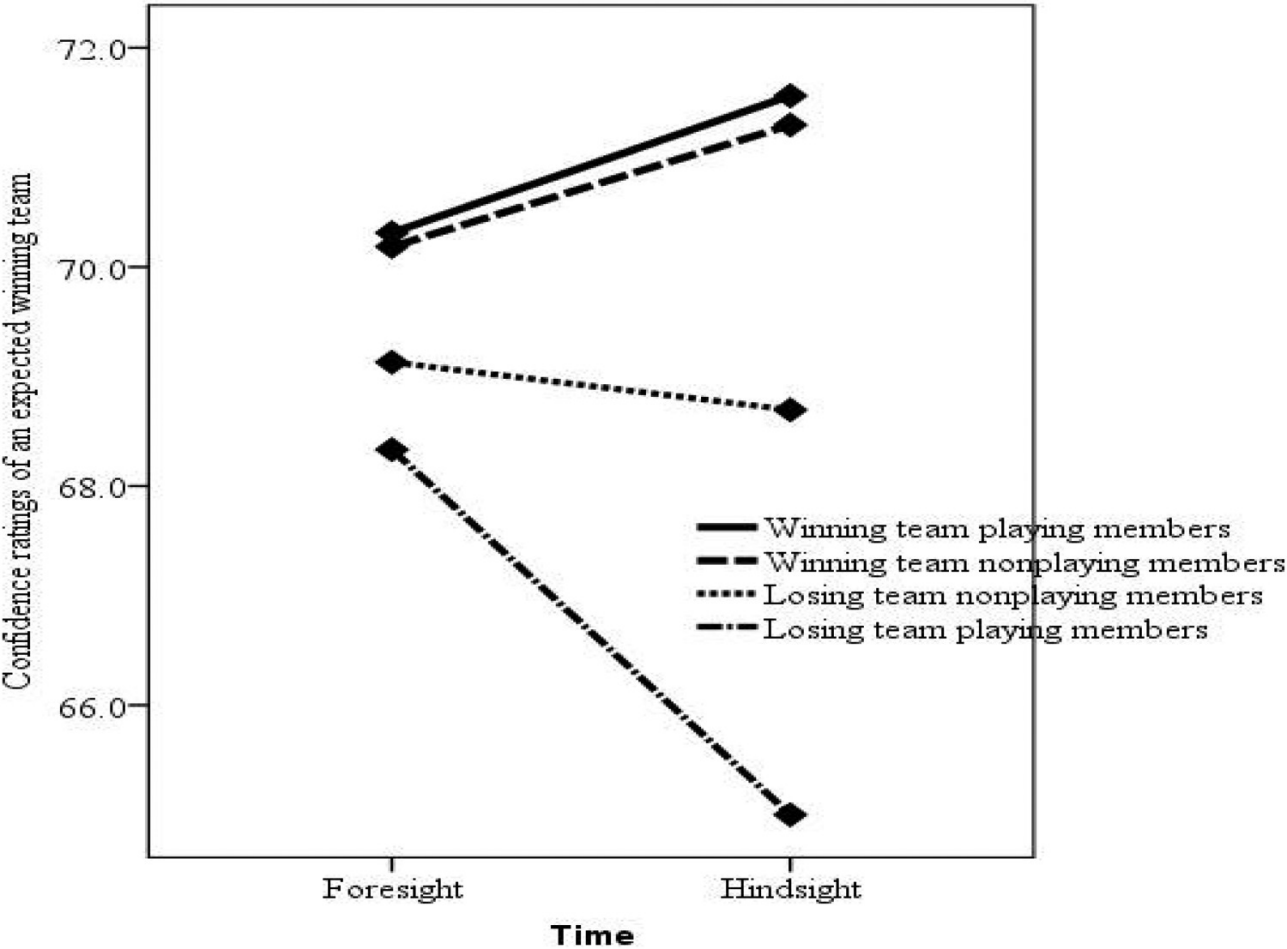
Positive effects of physical exercise on cognitive function across the lifespan.

**Figure 2:**
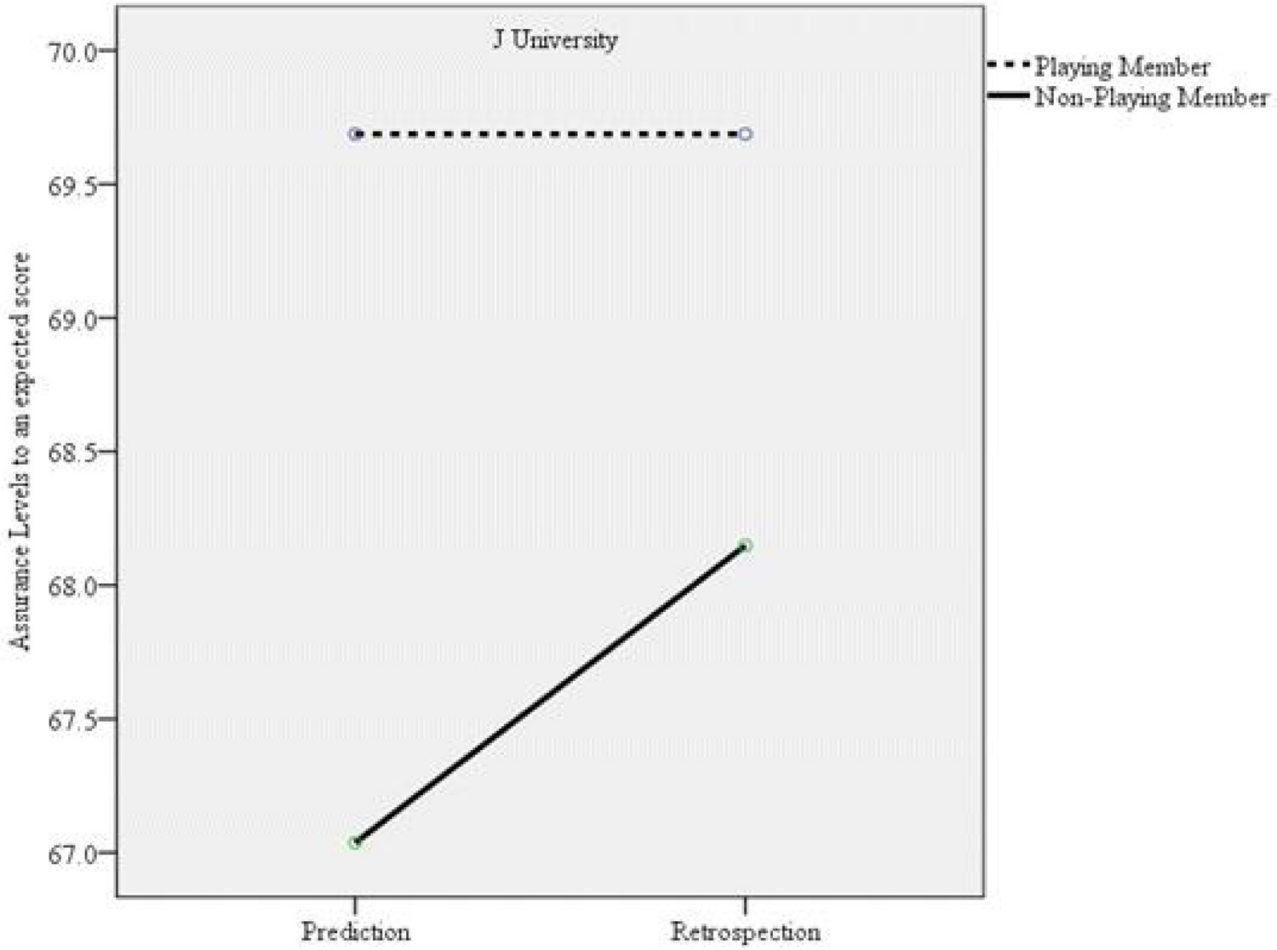
Interaction effect between the confidence levels for the expected match score between J university (winning team) playing members and nonplaying members.

An interaction effect between the time factor and the team and player factors was significant, *F*(3, 80) = 3.96, *p* = .011, = .129 (see Table 3). The effect size is presented using Cohen's partial eta squared. Cohen (39) suggested the following conventions for partial eta squared interpretations: small (.01), medium (.06), and large (.14) effect sizes. Based on Cohen's guidelines, the interaction effect had a medium-large effect size. Interaction graphs were generated to understand the general nature of the interaction and are presented in Figure 1. The interaction graphs show that based on the time factor, the winning team members increased their confidence ratings for the expected winning team while losing team members decreased their confidence ratings. Figure 2 shows losing team playing members had the largest change in confidence ratings between foresight and hindsight. This finding was further confirmed for losing team playing members by a paired sample t-test that showed a significant mean difference, *t*(17) = 2.38, *p* = .029, *d* = .47, between foresight (M = 68.33, SE = 1.67) and hindsight (M = 65.00, SE = 1.67). Paired sample t-tests between foresight and hindsight for winning team playing members, *t*(15) = −1.00, *p* = .333, winning team nonplaying members, *t*(26) = −1.363, *p* = .185, and losing team nonplaying members, *t*(22) = .569, *p* = .575, were not significant.

**Figure 3:**
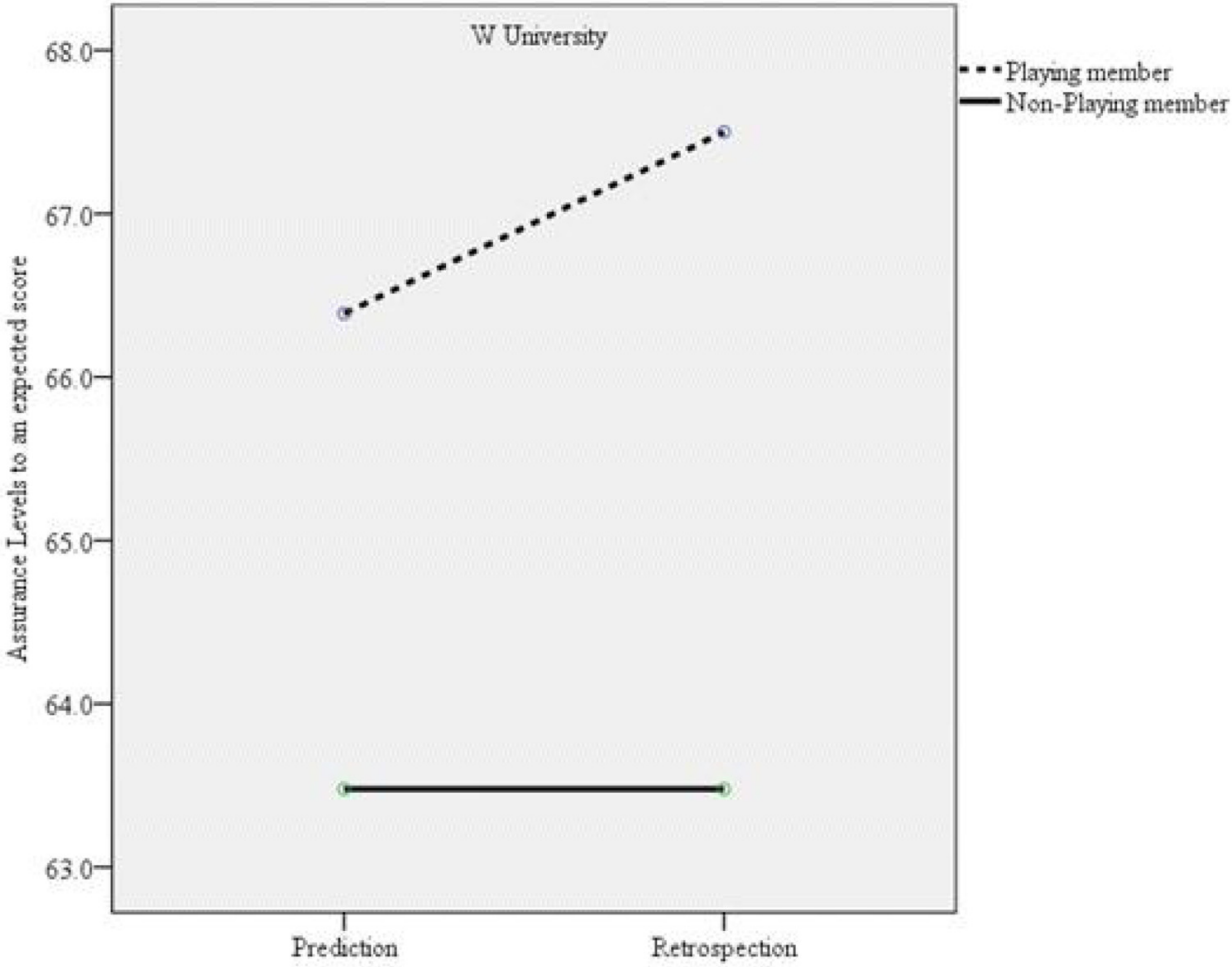
Interaction effect between the confidence levels for the expected match score between W university (losing team) playing members and nonplaying members.

The interaction effect was further examined by follow-up planned contrasts. Planned contrasts revealed that there was a statistically significant difference between winning team members and losing team members, *t*(80) = 2.28, *p* = .026, *r* = .247. More specifically, the winning team playing members significantly increased their confidence ratings compared with the losing team playing members, *t*(80) = 2.07, *p* = .042, *r* = .223. The winning team nonplaying members also showed a significant increase compared to the losing team playing members, (80) = 2.26, = .029, = .244 (See Figure 3). In the planned contrasts analysis, no significant difference was identified for playing and nonplaying members, (80) = −0.765, = .447; for playing and nonplaying members of the winning team, (80) = .104, = .918; for playing and nonplaying members of the losing team, (80) = −1.187, = .239; for winning team playing members and losing team nonplaying members, (80) = 1.034, = .304; or for nonplaying members of both the winning and losing teams, (80) = 1.071, = .287.

**Figure 4:**
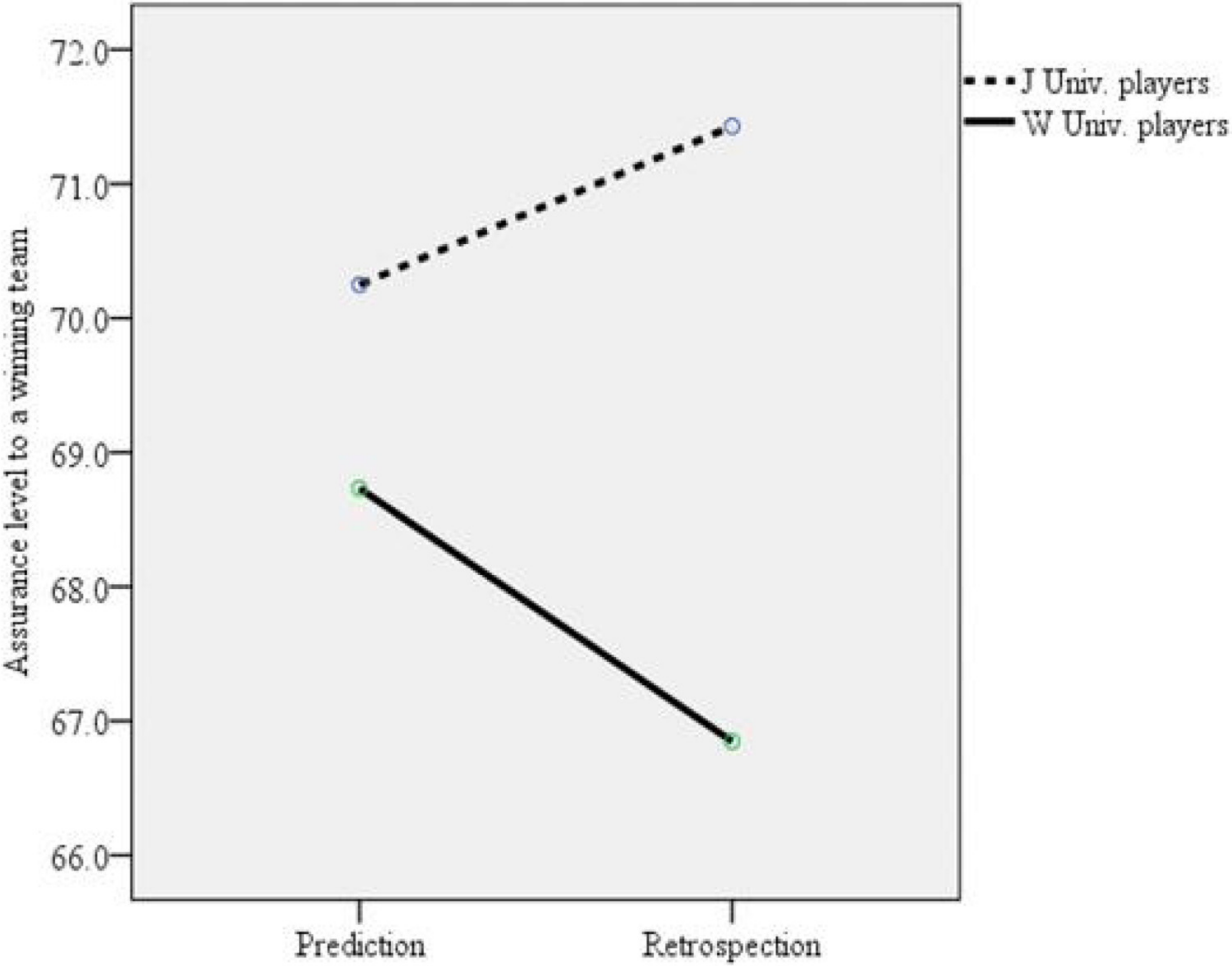
Interaction effect between the confidence levels for the expected winning team between J university (winning team) players and W university (losing team) players.

To further examine the occurrence of hindsight, one-way ANOVAs for foresight and hindsight were conducted separately. If a between-group effect was not identified for foresight but was observed for hindsight, this would indicate that participants experienced hindsight after the soccer match based on their assigned groups. One-way ANOVA for foresight revealed a nonsignificant between-group effect, (3, 80) = .401, = .753, = .148; however, one-way ANOVA for hindsight revealed a significant between-group effect, *F*(3, 80) = 4.319, *p* = .007, = .325. In the planned contrast analyses for the one-way ANOVA for hindsight, there were significant differences between winning team members vs. losing team members, *t*(80) = 3.213, *p* = .002, *r* = .338; winning team playing members vs. losing team playing members, *t*(80) = 2.984, *p* = .004, *r* = .316; and winning team nonplaying members vs. losing team playing members, *t*(80) = 3.233, *p* = .002, *r* = .339. For the hindsight measurements, there were no significant differences between playing vs. nonplaying members, (80) = −1.203, = .233; winning team playing members vs. nonplaying members, (80) = .132, = .895; losing team playing members vs. nonplaying members, (80) = −1.835, = .070; winning team playing members vs. losing team nonplaying members, (80) = 1.376, = .173; or winning team nonplaying members vs. losing team nonplaying members, (80) = 1.432, = .156. Effect sizes for t-tests, ANOVAs, and planned contrasts are presented using Cohen's *d*, omega (Ω), and *r*, respectively. Based on the guidelines provided by previous studies (39–41), the identified significant differences had modest to large effect sizes (See Figure 4).

## Discussion

Heuristics are usually used to analyze and evaluate current phenomenon, which can lead to fallacies because the foundation of heuristics is based on past experiences that may not be applied to current situations. This process can also lead people to believe that they are capable of explaining a number of current situations spontaneously and thoroughly. However, these explanations may be inferred from illogical deductions, resulting in biased reconstruction explanations (42). In contrast, the foundation of hindsight bias is based on the knowledge of present outcomes that people use to analyze and understand past situations. This hindsight bias can be used as a description of the systematic differences between predictive and retrospective judgments and can explain why retrospective judgments are influenced by outcomes (43). In this study, when team and player factors were accounted for, there were significant differences between foresight and hindsight in terms of the confidence ratings for the expected winning team, which shows that the participants' retrospective judgments were influenced by the match outcome based on their assigned groups. This finding was further confirmed by ANOVA; one-way ANOVA for foresight did not show significant differences in confidence ratings based on assigned groups, while for hindsight, significant differences were found among the assigned groups. Figure 1 presents the general tendency that when an event actually occurred, the recalled confidence ratings were larger than the original estimates, and when an event did not happen, the recalled confidence ratings were smaller, although only the losing team playing members showed statistically significant differences. These findings indicate that hindsight bias is found overall in our real-world outcomes, as the levels of confidence ratings reflected the match outcome.

It was expected that for a negative match outcome, the losing team playing members would show less hindsight bias because they were likely responsible for the negative outcome and would employ a self-defensive process, thus producing less hindsight bias. However, this finding was not seen in current study, as playing members showed significant differences in their confidence ratings between foresight and hindsight while nonplaying members did not. One possible cause for this unexpected finding is that the match outcome was probably determined by a penalty kick, which could have led to the low outcome controllability of the playing members who may have blamed the referee of the match for their loss. As a result, playing members considered the match outcome inevitable and did not feel the beginnings of a self-defensive process. Rather, they used retroactive pessimism and showed greater hindsight bias. This finding is consistent with a previous finding showing that even for uncontrollable events, self-defensive processes can occur when the events cannot cause a substantial threat to people themselves (20).

Another possible explanation for this unexpected finding is the magnitude of disappointment after losing to the other team. Disappointment is intense when a negative outcome is unexpected (23). Historically, the two teams in this study have been competitive with each other at similar levels of competition, and every participant expected that his team would win. Thus, it is clear that the losing team members felt a sense of disappointment after the match; playing members might have felt a greater disappointment in the match outcome than the nonplaying members might have. Thus, the magnitude of disappointment that was experienced differently by playing members and nonplaying members might yield different levels of hindsight bias. This notion is further supported by Tykocinski (22), which argues that people use retroactive pessimism as a means of coping with disappointment and that people prefer to consider a very disappointing outcome inevitable because “***an inescapable failure might be easier to digest than a failure that could have been easily avoided***” (p. 381). Thus, retroactive pessimism is a possible choice for playing members of the losing team, thus producing greater hindsight bias.

We originally expected that the winning team members would show greater hindsight bias because previous finding indicated that people show hindsight bias for their own good performance or a competing team's failure but no bias for their own failures or a competing team's good performance (44). However, there was no significant increase in the recalled confidence ratings in the current study, although trends were apparent, as presented in Figure 1.

Among members of the winning team, those who did not participate in the match exhibited an increase in their confidence regarding the accuracy of their predicted match score. In contrast, those who actively participated in the game did not demonstrate a similar increase in confidence. This pattern suggests that the hindsight bias observed in playing members is unlikely to be explained by differences in expertise between playing and nonplaying members. Musch and Wagner (51) noted “***to the extent that experts are better able to reliably recall their original judgment, reconstruction processes are rendered unnecessary, which should result in a smaller bias***” (p. 67). Similarly, Shanteau (52) reported that experts can process an enormous amount of information within a limited time when compared to novices. So, the decisions made by experts are akin to rational decision making and less similar to hindsight bias. In this study, playing members possibly had better quality and more knowledge about their match than the nonplaying members. Thus, nonplaying members were more susceptible to hindsight bias.

Pezzo (30) stated that “***relatively few studies have used self-relevant outcomes so we don't know much about their contribution***” (p. 668). Self-relevant *real-world* outcomes have rarely been used in hindsight bias research, and, to the best of our knowledge, no relevant published literature in sports psychology has addressed self-related motivation processes of hindsight bias in a real-world design. An account of the generalizability of real-world outcomes is of value, and respondents in this study were asked to recall their predictions four days after they made their initial predictions. In many laboratory studies, retrospections are measured within an hour of predictions (30). In this regard, the current study is unique and may contribute to this field. Admittedly, it is still difficult to randomly assign outcomes in a real-world design. However, Renner (45) argued that the benefits of employing real-world events sometimes outweigh nonrandomly assigned outcomes if the random assignment is ethically difficult or otherwise impossible. Future research addressing the self-related motivational aspects of hindsight bias in sports should consider laboratory settings to examine this phenomenon.

## What does this article add?

The influence of the self-related motivational aspects of hindsight bias in sports is largely unexplored, although social and cognitive psychologists have proposed that hindsight bias hinders people from learning (3) and leads people to make overly risky decisions (46). In this regard, this article exploring hindsight bias in soccer athletes using outcome controllability can contribute to this field. Since hindsight bias is rarely examined in a self-relevant real-world setting, the results presented in the current study provide valuable information. Given that athletes are not free from hindsight bias, which can make coaching less effective, restricting athletes from outcome knowledge, the main cause of hindsight bias, can be a training strategy for coaches. For example, it could be worth trying to not inform sprinters of their 100-m sprinting times for a period of time. The magnitude of disappointment, as mentioned above, can be associated with athletes' hindsight bias, but this relationship was not empirically examined and could be a future research topic. Furthermore, given that the level of expertise can influence hindsight bias, comparing hindsight bias between elite players and recreational players should be of great interest. Finally, individual differences, such as cognitive style (47) and ego involvement (48), can influence sports participants' hindsight bias. There is a dearth of hindsight bias research that accounts for individual differences, and examining these associations could be a promising and important research direction.

## Data Availability

The raw data supporting the conclusions of this article will be made available by the authors, without undue reservation.
